# From gene-specific to function-specific risk stratification in long QT syndrome Type 2: implications for clinical management

**DOI:** 10.1093/europace/euad035

**Published:** 2023-03-01

**Authors:** Lia Crotti

**Affiliations:** Istituto Auxologico Italiano IRCCS, Center for Cardiac Arrhythmias of Genetic Origin and Laboratory of Cardiovascular Genetics, Via Pierlombardo 22, 20135, Milan, Italy; Department of Medicine and Surgery, University of Milano-Bicocca, Via Cadore 48, 20900, Monza, Italy

## Abstract

**Graphical Abstract:**
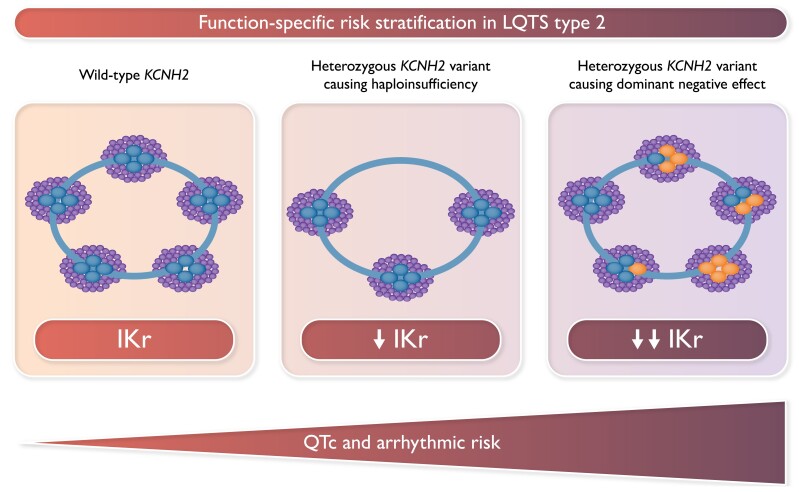
The graphical abstract represents three possible functional conditions. On the left is a cell with no disease-causing variant in the potassium voltage-gated channel subfamily H member 2 (*KCNH2*) gene, and therefore, potassium channels are composed by four wild-type (WT) subunits (blue small circles). In the middle panel is a cell with haploinsufficiency (HI): there are less potassium channels available, but all with WT subunits. On the right panel is a cell with a mutation causing a dominant-negative (DN) effect: channels are composed by a random mix of WT and mutant subunits (red small circles). The amount of rapid component of the delayed rectifier potassium current (IKr) is progressively decreasing going from WT to HI to DN with an opposite increase in QT interval corrected for heart rate (QTc) and arrhythmic risk.

The graphical abstract represents three possible functional conditions. On the left is a cell with no disease-causing variant in the potassium voltage-gated channel subfamily H member 2 (*KCNH2*) gene, and therefore, potassium channels are composed by four wild-type (WT) subunits (blue small circles). In the middle panel is a cell with haploinsufficiency (HI): there are less potassium channels available, but all with WT subunits. On the right panel is a cell with a mutation causing a dominant-negative (DN) effect: channels are composed by a random mix of WT and mutant subunits (red small circles). The amount of rapid component of the delayed rectifier potassium current (IKr) is progressively decreasing going from WT to HI to DN with an opposite increase in QT interval corrected for heart rate (QTc) and arrhythmic risk.

**This editorial refers to ‘Non-missense variants of KCNH2 show better outcomes in Type 2 long QT syndrome’, by Y. Wada *et al.*, https://doi.org/10.1093/europace/euac269.**

Long QT syndrome (LQTS) is certainly a model for all genetically transmitted arrhythmogenic diseases in terms of capability to use a genetic testing result to guide clinical management, as clearly highlighted in the last consensus document on the state of genetic testing for cardiac diseases.^1^ Such a result is the consequence of many studies that in the last 25 years successfully correlated genetic information with arrhythmic risk.^2–6^

As a matter of fact, it is very well known that some genetic subtypes are associated with a greater risk, i.e. variants in the calmodulin genes tend to manifest with life-threatening arrhythmias very early in life^2^ and the recessive Jervell Lange–Nielsen syndrome, especially when due to potassium voltage-gated channel subfamily Q member 1 (KCNQ1) mutations, is a severe form of the disease.^3^ Considering the three major genetic subtypes of LQTS [due to *KCNQ1*, potassium voltage-gated channel subfamily H member 2 (*KCNH2*), and sodium voltage-gated channel alpha subunit 5 (*SCN5A*)], risk is different according to the gene involved,^4^ the location of the variant in the protein,^4,5^ and in some cases the specific variant matters.^6^

The study by Aizawa *et al.*, published on the current issue of the journal, took a further step forward in the understanding on how genetic basis can influence the arrhythmic risk in the Type 2 variant of LQTS.^7^ Indeed, all the studies so far published^4,5,8^ correlated clinical severity with the location of the mutation in the protein or with the type of disease-causing genetic variant, but this is the first study that attempted to correlate the functional consequences of the variants with arrhythmic risk. The authors studied a population of 429 LQT2 patients, followed in two Japanese referral centres, carrying 178 unique *KCNH2* variants, 102 missense and 76 non-missense, the latter including non-sense, frameshift, or splicing variants.^7^ They divided all these variants into two major functional subgroups, those leading to haploinsufficiency (HI) and those causing a dominant-negative (DN) effect. For those not familiar with these terms, haploinsufficiency occurs when only a single copy of the gene is functional. Specifically, as the HERG potassium channel conducting rapid component of the delayed rectifier potassium current (IKr) current is composed by the assembly of four subunits all encoded by *KCNH2*, if the mutated protein is not able to coassemble with the wild-type (WT) one and to reach the cell surface, there will be less channels available, but all normally functioning. In contrast, a DN effect occurs when the mutant proteins coassemble with wild-types, reducing the activity of the co-expressed wild-type protein and therefore causing a more important reduction of Ikr current (see Graphical abstract). The authors attributed all non-missense variants (*n* = 76) to the group of HI as they may undergo non-sense-mediated mRNA decay (NMD), that is, a protective mechanism present in all eukaryotic cells, consisting in eliminating mRNA transcripts that contain premature stop codons to prevent the translation of aberrant proteins.^9^ Missense variants (*n* = 102) were classified into HI or DN group according to previously published functional data in 41 of them and according to predictions based on protein localization in the remaining 61.^7^ Specifically, the authors classified missense variants in the pore region and in the voltage sensor domain as variants with a presumed dominant-negative effect (pDN) and those in all other regions as presumed HI (pHI). The authors started comparing patients with non-missense variants, missense variants with established HI, and missense variants with established DN effect. Patients with non-missense variants and those in the established HI group had similar QT interval corrected for heart rate (QTc), significantly shorter than the one observed in patients in the established DN group (*P* < 0.001); in terms of probability of arrhythmic events, those in DN group showed the worst prognosis (*P* = 0.001). When the comparison was performed only in patients with pHI vs. those with pDN, there was a statistically significant difference in terms of both QTc and arrhythmic risk, with patients in the pDN group having the worst outcome. Finally, multivariate analysis confirmed DN status as an independent risk factor for cardiac events in the population under investigation.^7^

The findings of the present study are perfectly in line with the study by Moss *et al.*^5^ showing that patients with mutation in the pore region of the KCNH2 are at greater risk of cardiac events. Indeed, in that study, all mutations in the pore region were missense variants, therefore entering in the DN group according to the study by Aizawa *et al.* In contrast, an apparent discrepancy is present with the study by Shimizu *et al.*^8^ that showed that there was not a significant difference in event rates between missense and non-missense variants. Aizawa *et al.* tried to explain such a difference with two hypotheses, the first one being the different study protocol used, by which patients were censored at the time of therapy initiation in their study, but not in the one by Shimizu *et al.* The second hypothesis was the inclusion in their cohort of more benign cases, specifically school-age children who took routine ECG check-ups; however, even after the exclusion of these cases, their results did not change. A plausible alterative explanation could be a different representation of missense variants causing HI or DN effect in the two cohorts. Indeed, in the study by Aizawa *et al.*, 78% (186/239) of patients with missense variants were in the DN or pDN group,^7^ while in the study by Shimizu *et al.*, with all the limitations of extrapolating numbers from published data, the percentage could go from a minimum of 30% (260/860) considering only patients with missense variants in the pore region to a maximum of 35% (304/860) including also patients with missense variants in transmembrane S1–S4 that contains the voltage sensor domain.^8^ In any case, there is an overrepresentation of missense variants in the DN group in the study by Aizawa *et al.* compared to the study by Shimizu *et al.* (*P* < 0.0001) that could justify the different result obtained comparing missense vs. non-missense variant independently on their functional consequences.

So the strong message coming from the study lead by Minoru Horie is that what really matters, in terms of risk stratification, is the functional consequence of a given genetic variant impacting the degree of IKr reduction that is linked to QTc and arrhythmic risk (see *Graphical Abstract*).

These conclusions have implication for clinical management, because according to the known or presumed functional consequence of the disease-causing variant identified, integrated with clinical information, we could modulate our therapeutic approach. Indeed, on top of beta-blockers, for patients with a severe form of Type 2 LQTS, we could consider adding mexiletine therapy,^10^ after evaluation of its efficacy with an oral acute load, and/or left cardiac sympathetic denervation.^11^

Finally, also in terms of new treatments, we are moving from gene-specific to mechanism-specific treatment. Indeed, in an encouraging preliminary trial, we demonstrated that a drug combining lumacaftor and ivacaftor, already in clinical use for cystic fibrosis, significantly shortened QTc in two LQTS Type 2 patients with a trafficking defect.^12^ These clinical observations recapitulate *in vitro* results where lumacaftor rescued the trafficking defect in cardiomyocyte-derived induced pluripotent stem (iPS) cells from patients with Class 2 *KCNH2* mutations (those impairing trafficking).^13^ A second Phase 2 clinical trial is currently ongoing with the aim of testing the drug in 20 additional LQTS patients with a Class 2 variant (ClinicalTrials.gov Identifier: NCT04581408).

The field is clearly moving more and more towards a precision medicine approach guided also by genetic results, and the study by *Aizawa et al.* provided an important missing piece of the puzzle. What we are still lacking is the proper integration in a risk stratification algorithm of clinical variables, disease-causing variant with its functional consequences, and all known single nucleotide polymorphisms (SNPs) with a small effect size (known also as genetic modifiers)^14,15^ that act synergistically with the disease-causing variant to determine the final functional impairment of the ion channel, ultimately contributing to sudden cardiac death risk.
